# Supplementary material for: Peripersonal encoding of forelimb proprioception in the mouse somatosensory cortex
Source: Nat Commun. 2023 Apr 12;14:1866. doi: 10.1038/s41467-023-37575-w (PMC10097678; doi:10.1038/s41467-023-37575-w)
Supplement: Supplementary file 1 — Supplementary Information [file 41467_2023_37575_MOESM1_ESM.pdf]

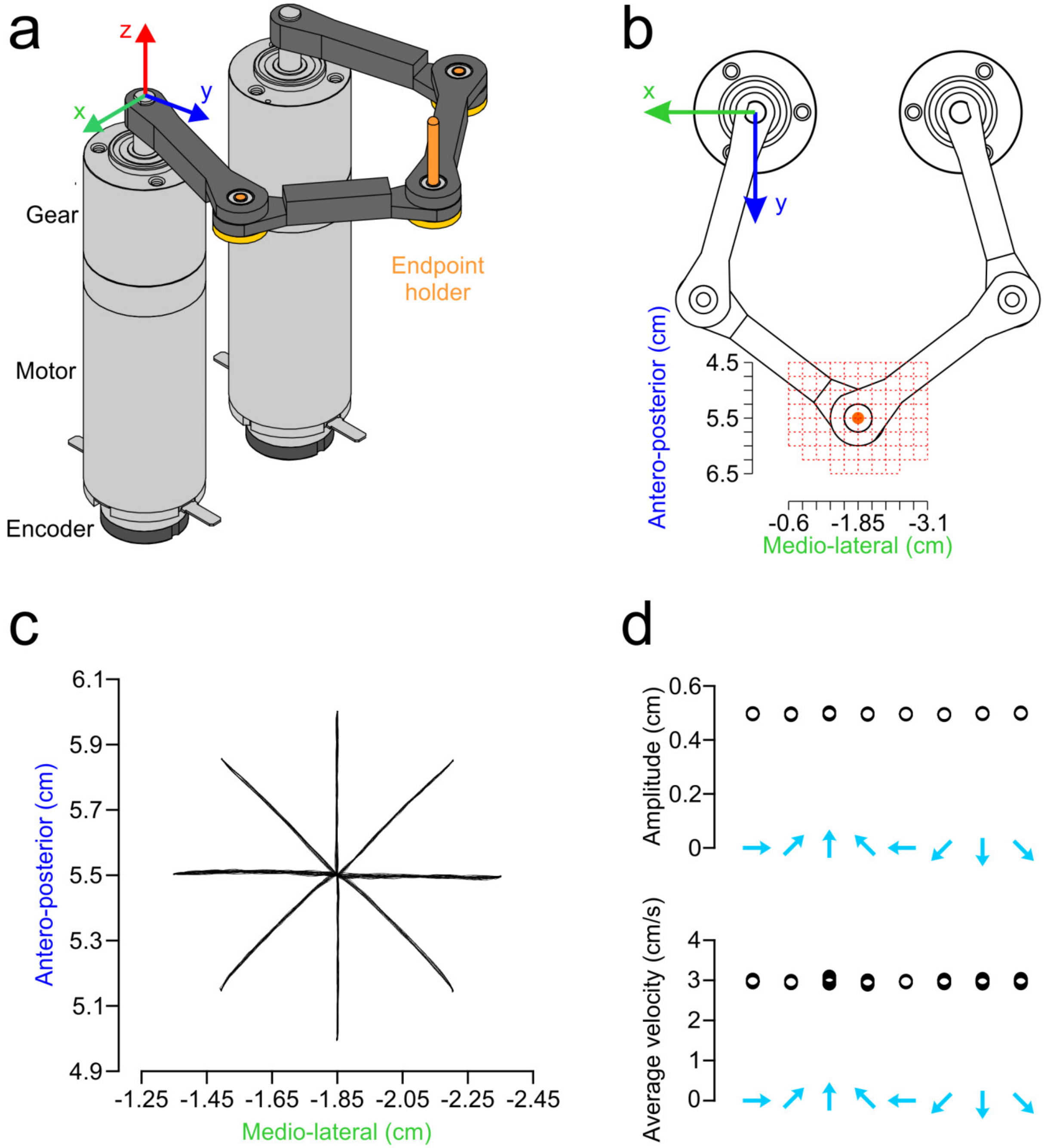

Supplementary Figure 1. Movement kinematics of the robotic manipulandum. a: CAD model of the robotic manipulandum showing the origin of the Cartesian coordinates. **b**: Top view of the manipulandum showing the workspace of forelimb movements (red grid). **c**: Superimposed 2D trajectories of the manipulandum's endpoint (raw unfiltered measurements with the optical encoders and sampled at 1 kHz, see Methods) for movements in the eight tested directions in an example session (17 to 23 trials per direction, 0.5 cm amplitude and 3 cm /s velocity). **d**: Measured 2D amplitudes and average velocities of the individual movements in A were highly consistent across the tested directions. Source data are provided as a Source Data file.

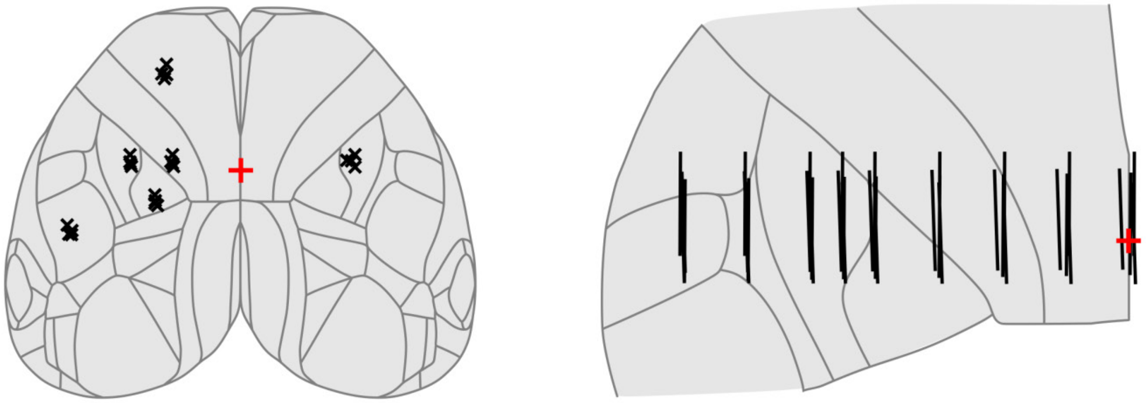

Supplementary Figure 2. Optogenetic stimulation sites. Stimulation coordinates of the optogenetic stimuli (Fig. 3d, f) registered to the surface projection of the Allen Mouse Brain Atlas indicate consistent targeting of the same areas across the 4 mice.

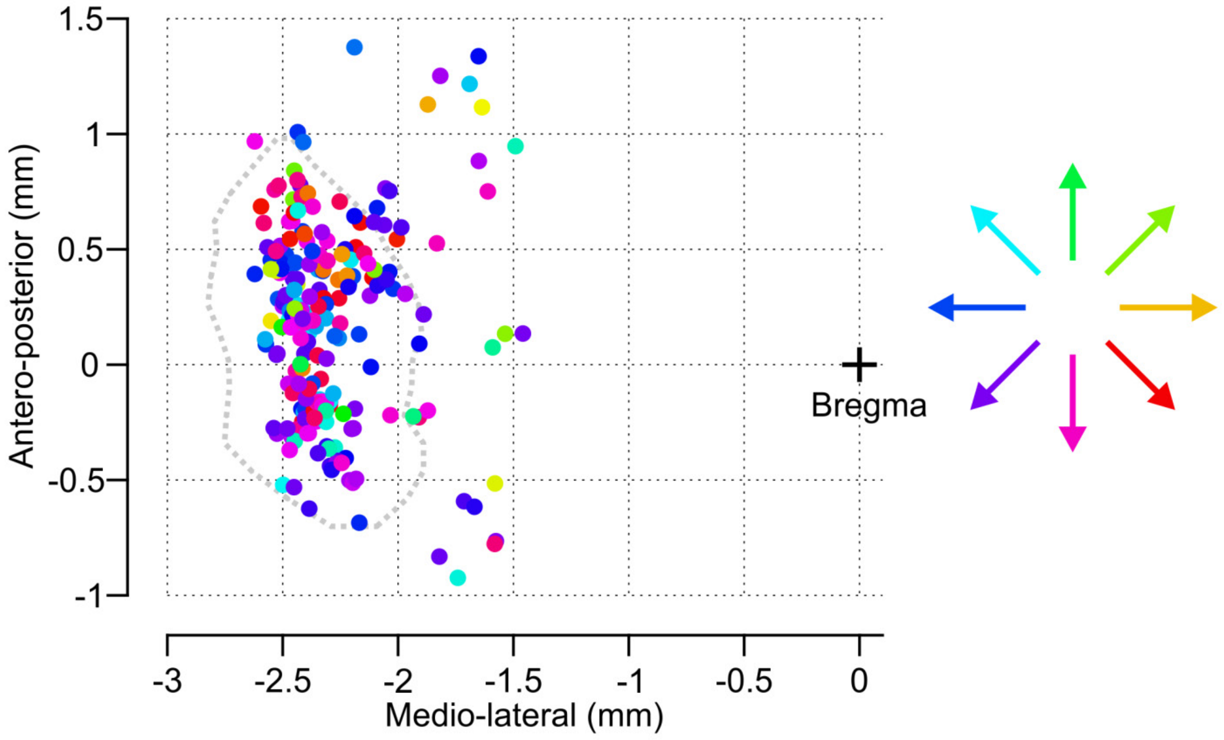

Supplementary Figure 3. Absence of directional topography in fS1. Antero-posterior and medio-lateral coordinates relative to bregma of directionally tuned proprioceptive neurons (N=225 neurons, 17 mice). The color code corresponds to the neuron's preferred direction. Gray dotted contour: limits of fS1 based on the mouse brain atlas[1]. Source data are provided as a Source Data file.

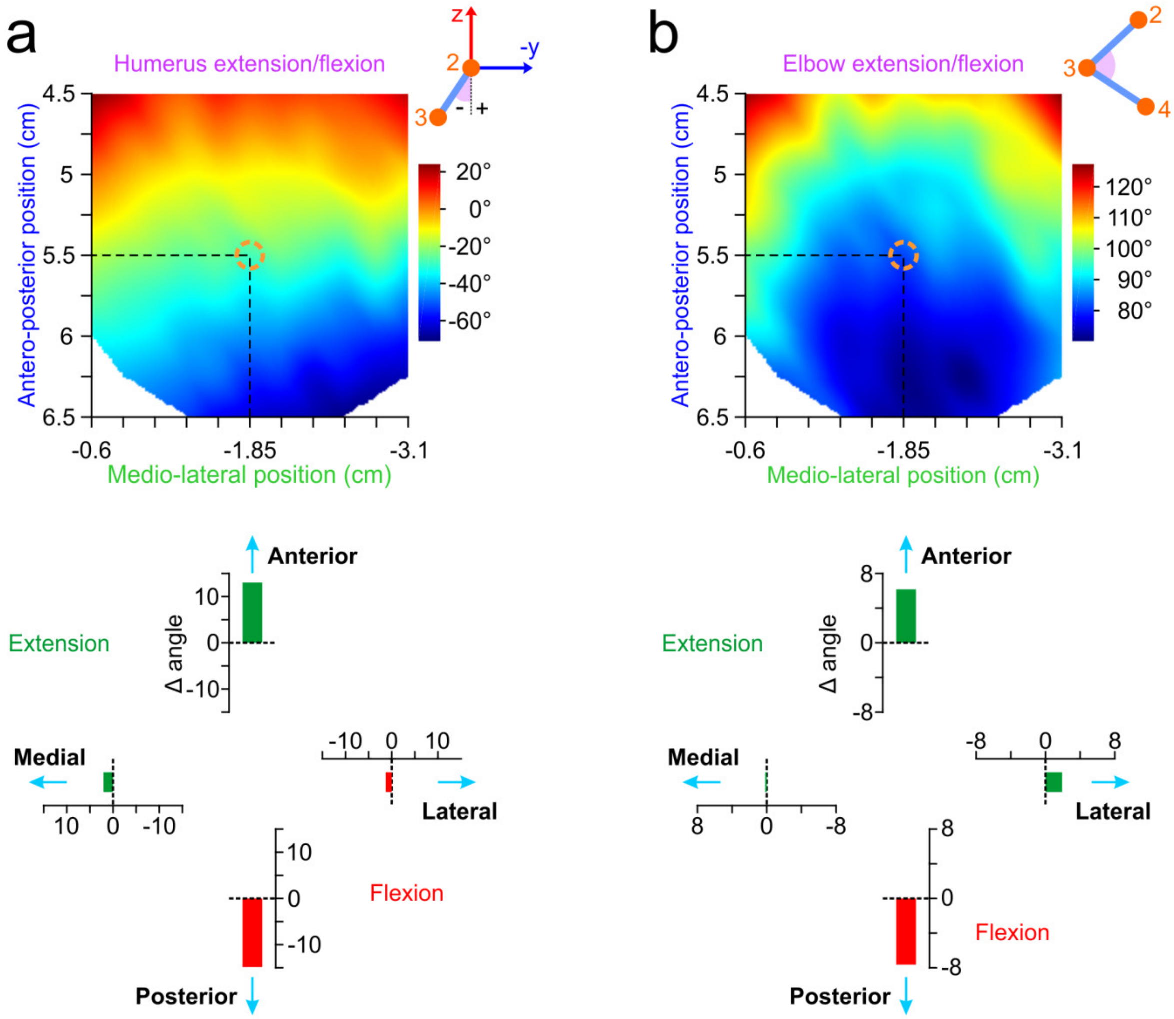

Supplementary Figure 4. Joint angles mapped onto the planar workspace of the robotic manipulandum. a: Top, humerus extension/flexion angle (color map, linear interpolation) mapped onto the planar workspace defined in Supplementary Fig. 1b (orange circle: manipulandum's home position). Bottom, changes (Δ angle measured from the map) in humerus flexion (negative values) or extension (positive values) as the limb is displaced by 4 mm in the medial, lateral, posterior and anterior directions. **b**: Same data as in a for elbow extension/flexion. Source data are provided as a Source Data file.

References

1 Paxinos, G. *The mouse brain in stereotaxic coordinates / George Paxinos, Keith B.J. Franklin*. (Academic, 2001).
