# Supplementary material for: Peripersonal encoding of forelimb proprioception in the mouse somatosensory cortex
Source: Nat Commun. 2023 Apr 12;14:1866. doi: 10.1038/s41467-023-37575-w (PMC10097678; doi:10.1038/s41467-023-37575-w)
Supplement: Supplementary file 2 — Description of Additional Supplementary Files [file 41467_2023_37575_MOESM2_ESM.pdf]

Description of Additional Supplementary Files

File Name: Supplementary Movie 1
Description: **Passive forelimb displacement task.** Example trial in the passive forelimb displacement task with a robotic manipulandum.

File Name: Supplementary Movie 2
Description: **Perceptual discrimination of proprioceptive stimuli.** Example lateral and medial displacement trials in the two-alternative forced choice (2AFC) discrimination task.

File Name: Supplementary Movie 3
Description: **3D tracking of joint positions.** 3D positions of 4 joints and the limb endpoint as well as their 2D projections were obtained for every manipulandum position in the tested planar workspace (red grid).
