# Supplementary material for: Peripersonal encoding of forelimb proprioception in the mouse somatosensory cortex
Source: Nat Commun. 2023 Apr 12;14:1866. doi: 10.1038/s41467-023-37575-w (PMC10097678; doi:10.1038/s41467-023-37575-w)
Supplement: Supplementary file 6 — Reporting Summary [file 41467_2023_37575_MOESM6_ESM.pdf]

nature portfolio

Corresponding author(s): Mario Prsa

Last updated by author(s): Feb 3, 2023

Reporting Summary

Nature Portfolio wishes to improve the reproducibility of the work that we publish. This form provides structure for consistency and transparency in reporting. For further information on Nature Portfolio policies, see our Editorial Policies and the Editorial Policy Checklist.

Statistics

For all statistical analyses, confirm that the following items are present in the figure legend, table legend, main text, or Methods section.

n/a	Confirmed	
☐	☒	The exact sample size (*n*) for each experimental group/condition, given as a discrete number and unit of measurement
☐	☒	A statement on whether measurements were taken from distinct samples or whether the same sample was measured repeatedly
☐	☒	The statistical test(s) used AND whether they are one- or two-sided<br>*Only common tests should be described solely by name; describe more complex techniques in the Methods section.*
☐	☒	A description of all covariates tested
☐	☒	A description of any assumptions or corrections, such as tests of normality and adjustment for multiple comparisons
☐	☒	A full description of the statistical parameters including central tendency (e.g. means) or other basic estimates (e.g. regression coefficient) AND variation (e.g. standard deviation) or associated estimates of uncertainty (e.g. confidence intervals)
☐	☒	For null hypothesis testing, the test statistic (e.g. *F*, *t*, *r*) with confidence intervals, effect sizes, degrees of freedom and *P* value noted<br>*Give P values as exact values whenever suitable.*
☒	☐	For Bayesian analysis, information on the choice of priors and Markov chain Monte Carlo settings
☒	☐	For hierarchical and complex designs, identification of the appropriate level for tests and full reporting of outcomes
☒	☐	Estimates of effect sizes (e.g. Cohen's *d*, Pearson's *r*), indicating how they were calculated

Our web collection on statistics for biologists contains articles on many of the points above.

Software and code

Policy information about availability of computer code

Data collection	Wide field calcium imaging data was acquired with the Image Proccessing Toolbox in Matlab2019b. Neuronal imaging data was collected with Scanimage 5.7_R1 software running on Matlab2019b. Behavioral data was collected with the Bpod_Gen2 (Sanworks) software running on Matlab2019b.
Data analysis	Ca2+ activity was extracted from the neuronal imaging data with the CaImAn-Matlab toolbox (Flatiron Institute, 2019 version) running on Matlab2019b. Spike rate was inferred with the OASIS deconvolution algorithm (Suite 2P toolbox, 2019 version). All other data analysis was performed with custom routines using standard Matlab functions and open source toolboxes (as detailed in the Methods). Custom scripts are available in the figshare repository: XX.

For manuscripts utilizing custom algorithms or software that are central to the research but not yet described in published literature, software must be made available to editors and reviewers. We strongly encourage code deposition in a community repository (e.g. GitHub). See the Nature Portfolio guidelines for submitting code & software for further information.

Data

Policy information about availability of data

All manuscripts must include a data availability statement. This statement should provide the following information, where applicable:

- Accession codes, unique identifiers, or web links for publicly available datasets
- A description of any restrictions on data availability
- For clinical datasets or third party data, please ensure that the statement adheres to our policy

The data generated in this study have been deposited in the XX database under accession code ZZ.

Human research participants

Policy information about studies involving human research participants and Sex and Gender in Research.

Reporting on sex and gender	The study did not involve human participants.
Population characteristics	The study did not involve human participants.
Recruitment	The study did not involve human participants.
Ethics oversight	The study did not involve human participants.

Note that full information on the approval of the study protocol must also be provided in the manuscript.

Field-specific reporting

Please select the one below that is the best fit for your research. If you are not sure, read the appropriate sections before making your selection.

☒ Life sciences ☐ Behavioural & social sciences ☐ Ecological, evolutionary & environmental sciences

For a reference copy of the document with all sections, see nature.com/documents/nr-reporting-summary-flat.pdf

Life sciences study design

All studies must disclose on these points even when the disclosure is negative.

Sample size	No statistical methods were used to predetermine sample size. For all tests, the used sample size yielded statistical powers in excess of 0.9.
Data exclusions	No data were excluded from analysis. For behavioral experiments, 4 additional animals were trained but did not reach the 75% correct criterion. They were excluded from any further experiments.
Replication	Anatomical tracing results were replicated in 3 animals. Behavioral measurements were replicated in 4 animals. All attempts at replication were successful. No other measure was taken to verify the reproducibility of the findings.
Randomization	No randomization was required as our study did not involve separating animals into control and experimental groups.
Blinding	Our study did not involve separating animals into control and experimental groups. Blinding during data collection with respect to group allocation is therefore not applicable for our study.

Reporting for specific materials, systems and methods

We require information from authors about some types of materials, experimental systems and methods used in many studies. Here, indicate whether each material, system or method listed is relevant to your study. If you are not sure if a list item applies to your research, read the appropriate section before selecting a response.

Materials & experimental systems

n/a	Involved in the study
	[x] Antibodies
[x]	[ ] Eukaryotic cell lines
[x]	[ ] Palaeontology and archaeology
	[x] Animals and other organisms
[x]	[ ] Clinical data
[x]	[ ] Dual use research of concern

Methods

n/a	Involved in the study
[x]	[ ] ChIP-seq
[x]	[ ] Flow cytometry
[x]	[ ] MRI-based neuroimaging

Antibodies

Antibodies used	Primary antibodies:<br>Rabbit anti-GFP, 1:1000, A-6455, Thermo Fisher;<br>Goat anti-tdTomato, 1:1000, AB8181-200, SICGEN;<br>Secondary antibodies:<br>Alexa Fluor 488 Donkey anti-Rabbit, 1:500, AB_2313584, Jackson Immuno Research;<br>Cy3 Donkey anti-Goat, 1:500, AB_2307351, Jackon Immuno Research
Validation	All primary antibodies are commercially available. The specificity of the primary antibodies was validated by the manufacturer. For details, see the manufacturer's website.

Animals and other research organisms

Policy information about studies involving animals; ARRIVE guidelines recommended for reporting animal research, and Sex and Gender in Research

Laboratory animals	Mice: C57BL/6, male, 12 to 15 week old<br>Mice: Thy1-GCaMP6f-GP5.17, male, 8 to 12 weeks old<br>Mice: VGAT-ChR2-eYFP mice, male and female, 8 to 12 weeks old<br>Mice: Rasgrf2-2A-dCre; Ai148(TIT2L-GC6f-ICL-tTA2)-D, male and female, 8 to 12 weeks old<br>Mice: PV-Cre; Ai32, male and female, 6 to 8 weeks old
Wild animals	The study did not involve wild animals.
Reporting on sex	The findings apply to male and female mice. Sex was not considered in the study design.
Field-collected samples	The study did not involve samples collected from the field.
Ethics oversight	All procedures were approved by and complied with the guidelines of the Fribourg Cantonal Commission for Animal Experimentation.

Note that full information on the approval of the study protocol must also be provided in the manuscript.
